# Glutaminase 1 plays a key role in the cell growth of fibroblast-like synoviocytes in rheumatoid arthritis

**DOI:** 10.1186/s13075-017-1283-3

**Published:** 2017-04-11

**Authors:** Soshi Takahashi, Jun Saegusa, Sho Sendo, Takaichi Okano, Kengo Akashi, Yasuhiro Irino, Akio Morinobu

**Affiliations:** 1 Department of Rheumatology and Clinical Immunology, Kobe University Graduate School of Medicine, 7-5-1, Kusunoki-Cho, Chuo-Ku, Kobe, 650-0017 Japan; 2 Department of Clinical Laboratory, Kobe University Hospital, 7-5-1, Kusunoki-Cho, Chuo-Ku, Kobe, 650-0017 Japan; 3 Division of Evidence-Based Laboratory Medicine, Kobe University Graduate School of Medicine, 7-5-1, Kusunoki-Cho, Chuo-Ku, Kobe, 650-0017 Japan

**Keywords:** Fibroblasts, Glutaminolysis, Glutamine, Metabolomics, Rheumatoid arthritis

## Abstract

**Background:**

The recent findings of cancer-specific metabolic changes, including increased glucose and glutamine consumption, have provided new therapeutic targets for consideration. Fibroblast-like synoviocytes (FLS) from rheumatoid arthritis (RA) patients exhibit several tumor cell-like characteristics; however, the role of glucose and glutamine metabolism in the aberrant proliferation of these cells is unclear. Here, we evaluated the role of these metabolic pathways in RA-FLS proliferation and in autoimmune arthritis in SKG mice.

**Methods:**

The expression of glycolysis- or glutaminolysis-related enzymes was evaluated by real-time polymerase chain reaction (PCR) and Western blotting, and the intracellular metabolites were evaluated by metabolomic analyses. The effects of glucose or glutamine on RA-FLS cell growth were investigated using glucose- or glutamine-free medium. Glutaminase (GLS)1 small interfering RNA (siRNA) and the GLS1 inhibitor compound 968 were used to inhibit GLS1 in RA-FLS, and compound 968 was used to study the effect of GLS1 inhibition in zymosan A-injected SKG mice.

**Results:**

GLS1 expression was increased in RA-FLS, and metabolomic analyses revealed that glutamine metabolism was increased in RA-FLS. RA-FLS proliferation was reduced under glutamine-deprived, but not glucose-deprived, conditions. Cell growth of RA-FLS was inhibited by GLS1 siRNA transfection or GLS1 inhibitor treatment. Treating RA-FLS with either interleukin-17 or platelet-derived growth factor resulted in increased GLS1 levels. Compound 968 ameliorated the autoimmune arthritis and decreased the number of Ki-67-positive synovial cells in SKG mice.

**Conclusions:**

Our results suggested that glutamine metabolism is involved in the pathogenesis of RA and that GLS1 plays an important role in regulating RA-FLS proliferation, and may be a novel therapeutic target for RA.

**Electronic supplementary material:**

The online version of this article (doi:10.1186/s13075-017-1283-3) contains supplementary material, which is available to authorized users.

## Background

Rheumatoid arthritis (RA) is a systemic autoimmune disease characterized by immune cell infiltration and proliferation in the synovium, leading to progressive joint destruction. Although the etiology of RA is not fully understood, recent evidence indicates that resident fibroblast-like synoviocytes (FLS) play a major role in initiating and driving RA [1]. RA-FLS exhibit anchorage-independent proliferation, lack contact inhibition in vitro, and can attach to and invade articular cartilage [1, 2]. Furthermore, RA-FLS survive autonomously in a tumor-like environment that is enriched with oxygen radicals, nitric oxide, and cytokines [3, 4]. In recent years, the development of biologic therapies that target pro-inflammatory cytokines and immune cells have greatly improved the treatment of RA; however, some patients remain resistant to these therapies. Thus, new therapies designed to suppress RA-FLS proliferation could replace or complement existing RA treatments, increasing the therapeutic efficacy for the nonresponding population.

Many inflammation and hypoxia-induced signaling pathways have profound effects on intracellular metabolism, leading to effects on cell growth and survival. Recent studies of cancer cell metabolism have revealed cancer-specific metabolic changes that have led to the identification of new therapeutic targets. Most cancer cells consume glucose at higher rates than normal cells, a phenomenon known as the Warburg effect [5]. Many studies have shown that glycolytic enzyme inhibitors efficiently inhibit tumor growth, both in vitro and in vivo, by shifting glucose metabolism from glycolysis to glucose oxidation. This shift results in the release of proapoptotic mediators and reduces malignant cell proliferation, thus eliminating actively growing tumor cells while leaving normal cells unaffected [6]. Glutamine is another carbon source that is important in cell growth and energy metabolism [7, 8]. Glutamine provides a major energy source for respiration and serves as a precursor for the synthesis of macromolecules such as nucleotides and proteins [9, 10]. In tumor cells, pyruvate generated from the glycolytic pathway is converted to lactate, rather than being used in the tricarboxylic acid (TCA) cycle. Although the requirement for mitochondrial ATP production is reduced in tumor cells, the demand for biosynthetic precursors and NADPH is increased [11]. To compensate for these changes and to maintain a functional TCA cycle, cancer cells often rely on elevated glutaminolysis [12–15]. Several cancer cells, such as HeLa and basal-type breast cancer cells [12, 16, 17], even favor glutamine over glucose as an energy source.

RA-FLS exhibit several tumor cell-like characteristics, but the role of glycolysis in RA-FLS proliferation is unclear. The microenvironment in RA inflamed joints is characterized by hypoxia and low nutrient concentrations [18–20]. A few reports have addressed the metabolic changes in RA-FLS. For example, metabolome analysis showed the different metabolomic profiling between RA-FLS and osteoarthritis (OA)-FLS [21]. In addition, the inhibition of glucose transporter 1, choline kinase, monocarboxylate transporter (MCT4), and 6-phosphofructo-2-kinase, or the activation of pyruvate dehydrogenase or administration of fructose 1,6-bisphosphate, have each been shown to inhibit RA-FLS proliferation and/or ameliorate inflammatory arthritis in mice [22–27]. The metabolic changes identified in RA-FLS represent novel mechanisms that contribute to the pathogenesis of rheumatic diseases, and the targeting of metabolic dysfunction may provide a new therapeutic approach for RA.

Since the roles of glycolysis and glutaminolysis in RA have not been thoroughly investigated, here we examined the role of these pathways in RA-FLS proliferation and the development of arthritis in SKG mice. The glutamine catabolism pathway is initiated by the conversion of glutamine to glutamate by glutaminase (GLS)1, and several studies have shown that GLS1 inhibition significantly suppresses cancer cell proliferation [28–31]. Here, we found that GLS1 expression was upregulated in RA-FLS, and that RA-FLS cell growth was decreased under glutamine-deprived, but not glucose-deprived, conditions. We also found that GLS1 inhibition suppressed RA-FLS proliferation and ameliorated inflammatory arthritis in SKG mice.

## Methods

### Mice

Female SKG mice were obtained from CLEA Japan (Tokyo, Japan). The mice were housed in the Kobe University animal facility at a constant temperature, with laboratory chow and water provided ad libitum. All animal protocols received prior approval by the institutional review board and all procedures were performed in accordance with the recommendations of the Institutional Animal Care Committee of Kobe University.

### Reagents and antibodies

Zymosan A (ZyA) was obtained from Sigma-Aldrich (St. Louis, MO, USA). Cytokines and platelet-derived growth factor (PDGF) were from R&D Systems (Minneapolis, MN, USA). Compound 968, 5-(3-bromo-4-(dimethylamino)phenyl)-2,2-dimethyl-2,3,5,6-tetrahydrobenzo[a]phenanthridin-4(1H)-one, was obtained from Calbiochem (La Jolla, CA, USA). Anti-glutaminase and anti-Ki-67 antibodies were obtained from Abcam (Cambridge, UK).

### Fibroblast-like synoviocytes and cell culture

Human studies were approved by the ethics committees of the Kobe University Hospital and conducted in accordance with the Declaration of Helsinki. Synovial tissue samples were obtained from RA and OA patients undergoing joint replacement surgery or synovectomy. The RA patients fulfilled the American College of Rheumatology 1987 criteria [32]. Collected synovial tissue samples were minced and incubated first with 4 mg/ml collagenase, and then with 0.05% trypsin (Difco, Detroit, MI, USA), as described previously [33]. The isolated cells were cultured in Dulbecco’s modified Eagle’s medium (DMEM) supplemented with 10% fetal bovine serum (FBS; GIBCO BRL, Palo Alto, CA, USA), 1% penicillin-streptomycin (Lonza Walkersville Inc., Walkersville, MD, USA), and 2 mM l-glutamine (GIBCO BRL). FLS cultures (used between passages 3–6) were maintained as previously described [33]. To determine the effects of glutamine or glucose deprivation, the FLS were cultured in DMEM with both l-glutamine and d-glucose (Wako, Osaka, Japan 044-29765), DMEM without l-glutamine (Wako 045-30285), or DMEM without d-glucose (Wako 042-32255), each of which was supplemented with 10% dialyzed FBS (Biowest, Nuaillé, France) and 1% penicillin-streptomycin.

### Metabolomic analyses

FLS intracellular metabolites (0.2–1.0 × 10^6^ cells) were analyzed by gas chromatography-mass spectrometry (GC/MS) and capillary electrophoresis-mass spectrometry (CE-MS; C-SCOPE, Human Metabolome Technologies Inc., Tsuruoka, Japan). GC/MS analysis was performed using a GC/MSQP2010 Ultra (Shimadzu Co., Kyoto, Japan) with a fused silica capillary column (CP-SIL 8 CB low bleed/MS; 30 m × 0.25 mm inner diameter, 0.25 μm film thickness; Agilent Technologies, Waldbronn, Germany), as described previously [34, 35]. 2-Isopropylmalic acid solution (Sigma-Aldrich) was added as an internal standard. The C-SCOPE analysis was performed as described previously [36]. 5-Hydroxy-N-methyl-tryptamine (Human Metabolome Technologies Inc.) was added as an internal standard.

### Real-time polymerase chain reaction

Total RNA was isolated using RNeasy (Qiagen, Hilden, Germany), and 1 μg of total RNA was reverse-transcribed with a QuantiTect reverse transcription kit (Qiagen). Quantitative real-time polymerase chain reaction (PCR) was performed using a QuantiTect SYBR Green PCR Kit (Qiagen) with an ABI Prism 9900 instrument (Applied Biosystems, Foster City, CA, USA), according to the manufacturer’s instructions. The primer pairs were from Qiagen and are shown in Additional file 1: Table S1. The mRNA levels were normalized to that of glyceraldehyde-3-phosphate dehydrogenase (GAPDH; QT01192646, Qiagen).

### Western blotting

Cell lysates were analyzed by Western blotting with anti-GLS and anti-β-actin antibodies (Sigma-Aldrich). The bound antibodies were visualized using a chemiluminescence reagent (Super Signal West Dura Extended Duration Substrate, Thermo Fisher Scientific, Waltham, MA, USA) following the manufacturer’s instructions. Immunoblot signals were quantified by densitometric scanning of the films, using ImageJ software.

### Cell viability assays

Cell viability was determined using a WST-8 Cell Proliferation Cytotoxicity Assay Kit (Dojindo Laboratories, Kumamoto, Japan). Cells were seeded into 96-well plates (1 × 10^4^ cells/well). After the culture period, the wells were pulsed with WST-8 for 3 h, and the optical density at 450 nm was measured with a microplate reader (Bio-Rad, Hercules, CA, USA).

### Cell proliferation assays

Cells were seeded into 96-well plates (1 × 10^4^ cells/well). After the culture period, cell proliferation was determined using a cell proliferation enzyme-linked immunosorbent assay (ELISA; BrdU; Roche, Basel, Switzerland) following the manufacturer’s instructions, and by measuring optical densities at 450 nm with a microplate reader (Bio-Rad).

### Arthritis induction and evaluation

Eight-week-old SKG mice were treated with 2 mg ZyA as previously described [37]. Briefly, ZyA suspended in saline was intraperitoneally injected on day 0, and arthritis developed 14 to 21 days later. Arthritis severity was assessed using a previously described scoring system (0 = no joint swelling, 0.1 = swelling of one digit joint, 0.5 = mild swelling of the wrist or ankle, and 1.0 = severe swelling of the wrist or ankle [38, 39]). The individual joint scores were totaled for each mouse.

### Histology and immunohistochemistry

The hind paws of the mice were removed, fixed in 4% paraformaldehyde, decalcified in EDTA, embedded in paraffin, and sectioned. The samples were then stained with hematoxylin and eosin, and histological evaluation was performed. Histological evaluation was performed using a previously described scoring system (0 = no inflammation, 1 = slight thickening of the synovial cell layer and/or the presence of some inflammatory cells in the sublining, 2 = thickening of the synovial lining, infiltration of the sublining, and localized cartilage erosions, and 3 = infiltration in the synovial space, pannus formation, cartilage destruction, and bone erosion [39]). Paraffin-embedded tissue sections (5 μm) were deparaffinized and hydrated with xylene and graded alcohols using a standard protocol, and incubated overnight with primary antibodies at 4 °C in a humidified chamber, and then rinsed and incubated with biotinylated secondary antibodies for 30 min at room temperature. The slides were developed using the ImmunoCruz™ ABC Staining System (Santa Cruz Biotechnology, Santa Cruz, CA, USA) and were counterstained with Mayer’s hematoxylin solution (Wako). Immunohistochemical staining of proliferating cells was performed with an anti-Ki-67 antibody. The Ki-67-positive cells in the lining of the synovium were quantified using ImageJ software.

### Treatment of SKG mice with compound 968

Compound 968 was dissolved in dimethyl sulfoxide (DMSO; Sigma-Aldrich) and administered intraperitoneally to SKG mice at 25 mg/kg three times per week from day 14 (before the onset of arthritis) to day 42 after ZyA injection. DMSO was used as a negative control.

### Statistical analysis

Results are expressed as the mean ± SEM. Statistical comparisons were performed using the Student’s *t* test, Mann-Whitney *U* test, and Welch’s *t* test, and two-way analysis of variance (ANOVA) using GraphPad Prism software as appropriate. *P* values less than 0.05 were considered statistically significant.

## Results

### Increased expression of mRNAs encoding HK2, MCT4, PDK1, and GLS1 in RA-FLS

**Fig. 1:**
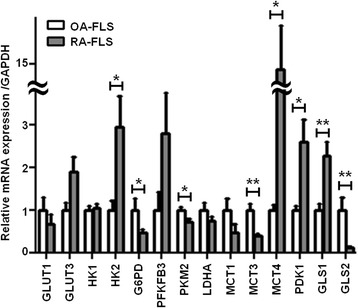
RA-FLS exhibit higher HK2, MCT4, PDK1, and GLS1 mRNA levels than OA-FLS. Glycolysis- and glutaminolysis-related mRNAs were examined in 12 OA-FLS and 19 RA-FLS by real-time PCR, and their levels were normalized to that of GAPDH mRNA. Each experiment was performed in triplicate. Bars indicate mean ± SEM. *P < 0.05, **P < 0.01, versus OA-FLS by Student’s *t* test. *G6PD* glucose-6-phosphate dehydrogenase, *GAPDH* glyceraldehyde-3-phosphate dehydrogenase, *GLS* glutaminase, *GLUT* glucose transporter, *HK* hexokinase, *LDHA* lactate dehydrogenase, *MCT* monocarboxylate transporter, *OA-FLS* fibroblast-like synoviocytes from osteoarthritis patients, *PDK* pyruvate dehydrogenase kinase; *PFK* 6-phosphofructo-2-kinase/fructose-2,6-bisphosphatase, *PKM* pyruvate kinase isozyme, *RA-FLS* fibroblast-like synoviocytes from rheumatoid arthritis patients

To determine which metabolic pathways are upregulated in RA-FLS, we compared the expression of 14 glycolysis- or glutaminolysis-related genes in RA-FLS to that in OA-FLS by real-time PCR. We found that the mRNA levels of hexokinase (HK)2, MCT4, pyruvate dehydrogenase kinase (PDK)1, and GLS1 were significantly higher in RA-FLS than in OA-FLS. mRNA levels of glucose transporter (G6PD), pyruvate kinase isozyme (PKM)2, MCT3, and GLS2 were significantly higher in OA-FLS than in RA-FLS (Fig. 1). The expression level of GLS2 was extremely low compared to GLS1, suggesting that GLS1 plays a major role in glutamine metabolism (Additional file 2: Figure S1).

### Upregulation of the glycolytic and glutaminolytic pathways in RA-FLS

**Fig. 2:**
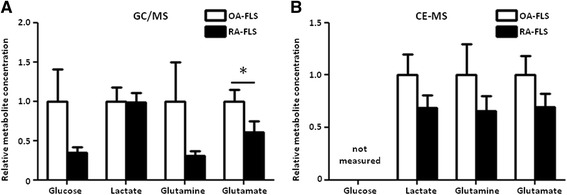
Glucose, glutamine, and glutamate are more highly consumed in RA-FLS than in OA-FLS. **a** Relative levels of intracellular metabolites in 7 OA-FLS and 11 RA-FLS were analyzed by GC/MS. **b** Relative levels of intracellular metabolites in 3 OA-FLS and 3 RA-FLS were analyzed by CE-MS. Bars indicate mean ± SEM. *P < 0.05 by Mann-Whitney *U* test. *CE-MS* capillary electrophoresis-mass spectrometry, *GC/MS* gas chromatography-mass spectrometry, *OA-FLS* fibroblast-like synoviocytes from osteoarthritis patients, *RA-FLS* fibroblast-like synoviocytes from rheumatoid arthritis patients

To further elucidate the altered metabolic regulation in RA-FLS, we assessed the intracellular metabolomic profiles of RA-FLS and OA-FLS using GC/MS and CE-MS. Both methods showed that the levels of glucose, glutamine, and glutamate tended to be lower in RA-FLS than in OA-FLS, suggesting that the glucose, glutamine, and glutamate consumptions were higher in RA-FLS (Fig. 2), although we did not find significant differences in the glutamine/glutamate ratio between OA-FLS and RA-FLS (Additional file 3: Figure S2). These results, together with the mRNA expression profiles (Fig. 1), indicated that both the glycolytic and glutaminolytic pathways are upregulated in RA-FLS.

### Importance of glutamine for RA-FLS proliferation

**Fig. 3:**
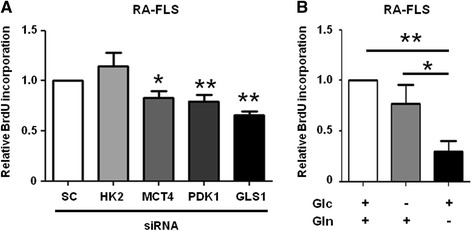
Glutamine is required for the proliferation of RA-FLS. **a** RA-FLS proliferation was determined using the BrdU assay 96 h after transfection with HK2, MCT4, PDK1, GLS1, or SC siRNA (*n* = 5). Each experiment was performed in quintuplicate. Bars indicate the mean ± SEM. *P < 0.05, **P < 0.01 versus SC by Student’s *t* test. **b** RA-FLS proliferation was determined using the BrdU assay 96 h after culturing in medium with both Glc and Gln, or in medium without Glc or Gln (*n* = 7). Each experiment was performed in quintuplicate. Bars indicate mean ± SEM. *P < 0.05, **P < 0.01. *Glc* glucose, *Gln* glutamine, *GLS* glutaminase, *HK* hexokinase, *MCT* monocarboxylate transporter, *PDK* pyruvate dehydrogenase kinase, *RA-FLS* fibroblast-like synoviocytes from rheumatoid arthritis patients, *SC* control scrambled, *siRNA* small interfering RNA

We next examined the roles of HK2, MCT4, PDK1, and GLS1 in RA-FLS proliferation. Smaill interfering RNA (siRNA) efficiency is shown in Additional file 4: Figure S3. The knockdown of MCT4, PDK1, or GLS1, but not HK2, significantly inhibited RA-FLS proliferation (Fig. 3a). Silencing of MCT4, PDK1, or GLS1 did not significantly increase or decrease interleukin (IL)-6 or matrix metalloproteinase (MMP)-3 production (Additional file 5: Figure S4). We then studied the requirement of glucose or glutamine for RA-FLS proliferation and found that the RA-FLS cell growth was significantly reduced under glutamine-deprived, but not glucose-deprived, medium conditions (Fig. 3b). Under the glutamine-containing medium condition, we found that RA-FLS proliferation was increased after PGDF stimulation, whereas under the glutamine-deprived medium condition we found that RA-FLS proliferation was not increased even after PDGF stimulation (Additional file 6: Figure S5). These results suggested that glutamine plays a more important role than glucose in RA-FLS proliferation.

### Upregulation of GLS1 in RA-FLS

**Fig. 4:**
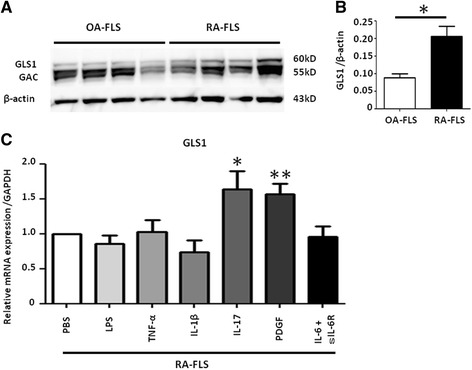
GLS1 is upregulated in RA-FLS. **a** GLS1 protein levels in 4 OA-FLS and 4 RA-FLS were determined by immunoblotting. GAC is a splicing variant of GLS1. **b** Protein levels were calculated by phosphorimager analysis. Bars indicate mean ± SEM. *P < 0.05 by Mann-Whitney *U* test. **c** RA-FLS were stimulated with LPS (1 μg/ml), TNF-α (10 ng/ml), IL-1β (2 ng/ml), IL-17 (20 ng/ml), PDGF (10 ng/ml), and IL-6 (100 ng/ml) and sIL-6R (100 ng/ml), or PBS for 24 h, and the GLS1 mRNA levels were analyzed by real-time PCR (*n* = 8). Each experiment was performed in triplicate. Bars indicate mean ± SEM. *P < 0.05, **P < 0.01, versus PBS. *GAC* Glutaminase C, *GAPDH* glyceraldehyde-3-phosphate dehydrogenase, *GLS* glutaminase, *IL* interleukin, *LPS* lipopolysaccharide, *OA-FLS* fibroblast-like synoviocytes from osteoarthritis patients, *PBS* phosphate-buffered saline, *PDGF* platelet-derived growth factor, *RA-FLS* fibroblast-like synoviocytes from rheumatoid arthritis patients, *sIL-6R* soluble IL-6 receptor, *TNF* tumor necrosis factor

Next, we evaluated the expression of GLS1, a key rate-limiting enzyme in glutaminolysis, in FLS. Western blot analysis revealed that the GLS1 expression was significantly higher in RA-FLS than in OA-FLS (Fig. 4a and b). We did not find upregulation of HK2, MCT4, or PDK1 in RA-FLS at a protein level. We then examined the effect of pro-inflammatory cytokines and growth factors implicated in the pathogenesis of RA on the GLS1 expression in RA-FLS. We found that IL-17 and PDGF significantly increased the GLS1 mRNA expression in RA-FLS, while lipopolysaccharide (LPS), tumor necrosis factor (TNF)-α, IL-1β, or IL-6 and soluble IL-6 receptor (sIL-6R) did not (Fig. 4c). We did not demonstrate upregulation of GLS1 in RA-FLS after IL-17 or PDGF stimulation at the protein level. IL-17 or PDGF stimulation did not significantly change the intracellular glutamine and glutamate levels in RA-FLS (Additional file 7: Figure S6).

### GLS1 inhibition suppresses RA-FLS proliferation

**Fig. 5:**
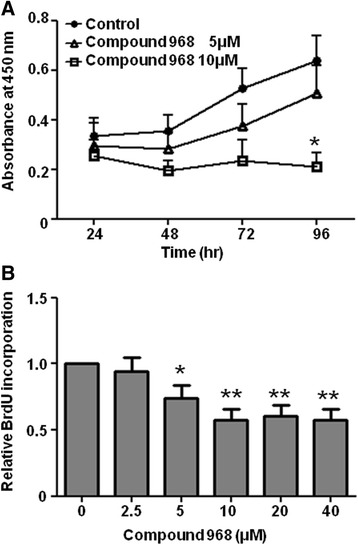
Compound 968, a GLS1 inhibitor, suppresses the proliferation of RA-FLS. **a** RA-FLS were cultured with or without compound 968 and quantified using the WST-8 assay (*n* = 3). Each experiment was performed in quintuplicate. Bars indicate mean ± SEM. *P < 0.05, versus control by Welch’s *t* test. **b** RA-FLS were cultured with various concentrations of compound 968 for 96 h. Cell proliferation was analyzed using the BrdU assay (*n* = 6). Each experiment was performed in quintuplicate. Bars indicate mean ± SEM. *P < 0.05, **P < 0.01, versus control

We next investigated the effect of compound 968, a GLS1 inhibitor, on RA-FLS cell growth. We demonstrated that 10 μM compound 968 significantly inhibited RA-FLS proliferation (Fig. 5a) in a dose-dependent fashion (Fig. 5b). These results further supported the notion that GLS1 plays an important role in synovial hyperplasia in RA.

### GLS1 inhibition ameliorates autoimmune arthritis in SKG mice

**Fig. 6:**
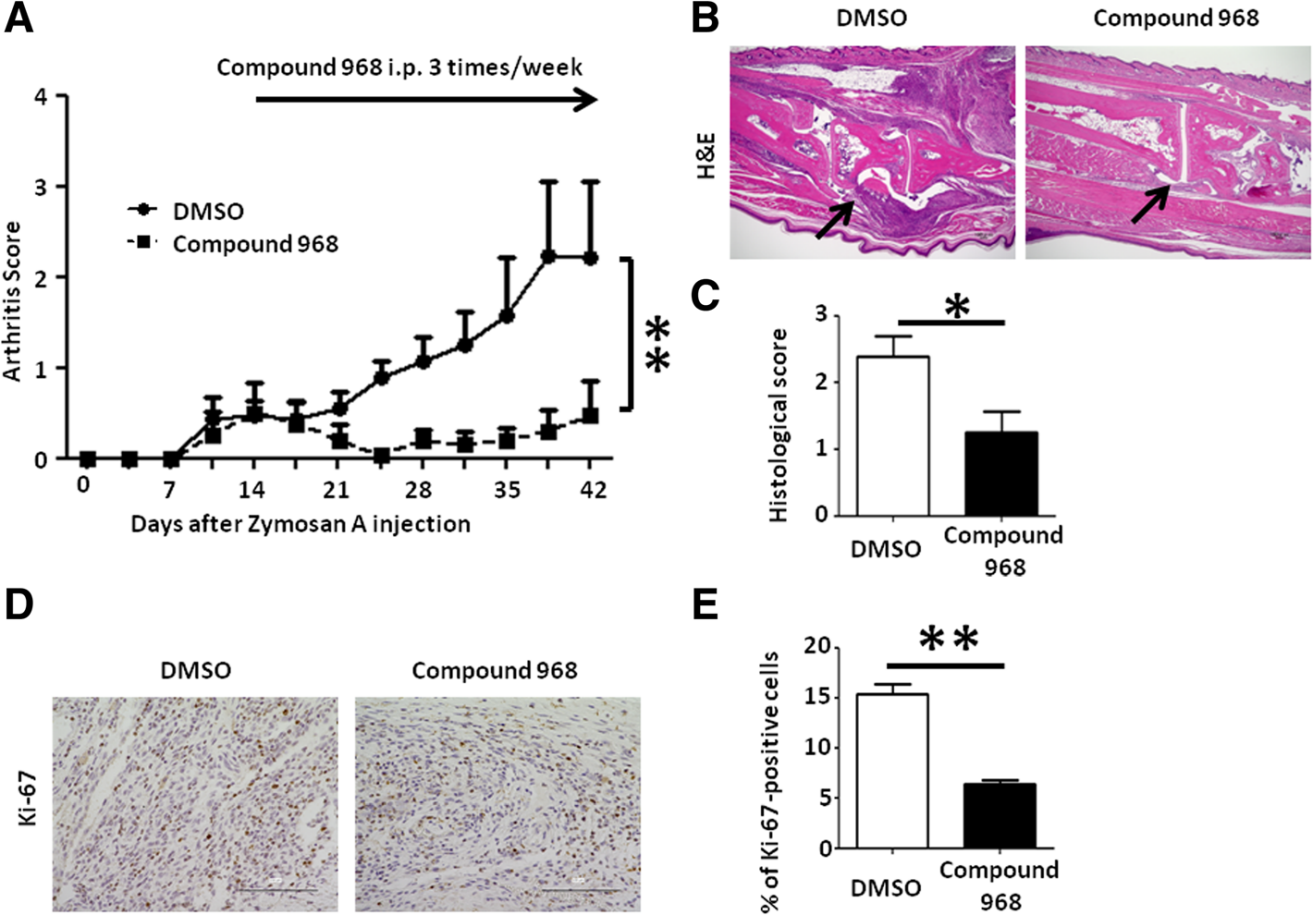
GLS1 inhibition ameliorates autoimmune arthritis in SKG mice. Fourteen days after ZyA injection, compound 968 or DMSO was injected intraperitoneally into SKG mice three times per week (compound 968, *n* = 4; DMSO, *n* = 5). **a** Clinical arthritis scores were determined up to 42 days after ZyA injection. **P < 0.01 by two-way ANOVA using GraphPad Prism software. **b** Hind paws of compound 968- or DMSO-treated SKG mice were assessed for histopathological changes 42 days after ZyA injection. *Arrows* indicate the lining of the synovium. H&E original magnification × 40. **c** Histological arthritis scores were determined in the hind paws of compound 968- or DMSO-treated SKG mice. *P < 0.05 by Student’s *t* test. **d** Immunohistochemical analysis of Ki-67 expression in the right hind paws of compound 968- or DMSO-treated SKG mice on day 42; original magnification × 400. **e** Quantification of Ki-67 staining from (**d**). Bars indicate mean ± SEM. **P < 0.01 by Student’s *t* test. *DMSO* dimethyl sulfoxide, *H&E* hematoxylin and eosin, *i.p.* intraperitoneally

Next, we investigated the therapeutic effect of compound 968 on autoimmune arthritis in the SKG mice, an animal model of RA. We injected compound 968 (25 mg/kg) three times per week from day 14 to 42 after ZyA injection. We showed that the arthritis severity in the compound 968-treated group was significantly decreased compared to the DMSO-treated group (Fig. 6a). We also examined the compound 968-treated and DMSO-treated SKG mice histologically. Arthritis histological scores were significantly lower in the compound 968-treated mice compared to DMSO-treated mice (Fig. 6b and c). Furthermore, immunohistochemical analysis revealed that compound 968 treatment significantly reduced the number of proliferative Ki-67-positive synovial cells (Fig. 6d and e), suggesting that GLS1 inhibition directly affects the cell-cycle progression of RA-FLS. We also measured the number of RORγt-expressing CD4^+^ Th17 cells and FoxP3-expressing CD4^+^ regulatory T (Treg) cells in the spleens from compound 968-treated and DMSO-treated SKG mice. There was no difference in the number of Th17 cells between the two groups, and the number of Treg cells was decreased in the compound 968-treated group, probably due to the amelioration of arthritis (Additional file 8: Figure S7). These results suggested that the influence of GLS1 inhibition on immune cells was minimal.

## Discussion

In this study, we showed that GLS1 expression and glutamine and glutamate consumption were higher in RA-FLS than in OA-FLS. We also showed that glutamine deprivation or GLS1 inhibition suppressed RA-FLS proliferation. Finally, we showed that the administration of a GLS1 inhibitor ameliorated inflammatory arthritis in a mouse model of RA by suppressing FLS proliferation. Taken together, this is the first report to show the importance of glutaminolysis in the pathogenesis of RA.

We found that mRNAs encoding HK2, MCT4, and PDK1 were more highly expressed in RA-FLS than in OA-FLS, although we could not find demonstrate the upregulation of these enzymes at the protein level. We also showed that levels of glucose, glutamine, glutamate, and lactate tended to be lower in RA-FLS than in OA-FLS. The reduction of the lactate level seen in CE-MS may be due to the elevation of the MCT4, which exports intracellular lactate to the extracellular space. These results, together with the mRNA expression profile and the intracellular metabolomic profiles, indicate that both the glycolytic and glutaminolytic pathways are upregulated in RA-FLS, and are consistent with previous reports showing that the inhibition of HK2, MCT4, or PDK1 suppresses RA-FLS proliferation and arthritis in a mouse model [22, 24, 26]. In addition, we found that silencing of MCT4, PDK1, or GLS1 did not significantly affect IL-6 or MMP-3 production from RA-FLS, suggesting that silencing of glycolytic or glutaminolytic enzymes may have little impact on cytokine production of these cells. Our findings that GLS1 plays key roles in RA-FLS proliferation and in a mouse model of arthritis further confirm that metabolic enzymes are involved in the pathogenesis of RA.

Cancer cells or highly proliferative cells exhibit a “glutamine addiction” phenotype [12]. To determine whether RA-FLS exhibit a similar phenotype, we investigated the effect of glucose or glutamine deprivation on RA-FLS proliferation. We found that RA-FLS proliferation was more dependent on glutamine than on glucose (Fig. 3b), and that GLS1 knockdown, but not HK2 knockdown, inhibited RA-FLS proliferation (Fig. 3a), consistent with a “glutamine addiction” phenotype of RA-FLS. Furthermore, we found that PDGF stimulation did not enhance the proliferation of RA-FLS under the glutamine-deprived condition. Notably, Colombo et al*.* [40] showed that HeLa cells, which strongly depend on glutamine for proliferation [14], transit through the S and enter the G2/M phase in the absence of glucose, but fail to enter G2/M in the absence of glutamine, suggesting that glutamine is critical for the entrance of highly proliferative cells into G2/M. They also showed that GLS1 is a substrate for the ubiquitin ligase anaphase promoting complex/cyclosome (APC/C)-Cdh1 in some cells [40, 41]. APC/C-Cdh1 inactivation is necessary for S phase initiation, and APC/C-Cdh1 substrates are targeted for degradation through specific recognition motifs such as the KEN box. These previous findings are consistent with our result that compound 968 treatment of SKG mice significantly decreased the number of Ki-67-positive synovial cells.

Although the regulation of GLS1 in cancer cells is not well understood, several studies indicate a functional link between the oncogene *MYC* and glutamine metabolism. Bush et al*.* showed that three of the five enzymes involved in glutamine metabolism are directly regulated by MYC at the transcriptional level [42]. c-MYC, which is known to stimulate cell proliferation, transcriptionally represses miR-23a and miR-23b, which are repressors of GLS1 expression. In addition, Gao et al*.* reported that GLS1 levels are closely correlated with MYC expression levels [16]. Studies also showed that the MYC expression is elevated in RA-FLS [43–45], raising the possibility that GLS1 expression is upregulated by MYC in these cells.

Our finding that PDGF or IL-17 augmented the GLS1 expression in RA-FLS at a mRNA level further supported the notion that glutaminolysis is important in RA-FLS metabolism, although we did not confirm the upregulation of GLS1 at the protein level nor the change in the intracellular glutamine and glutamate levels after IL-17 or PDGF stimulation. PDGF is a well-known growth factor for RA-FLS, and induces MYC expression [46]. IL-17 is also abundant in the rheumatoid synovium and contributes to RA pathogenicity by inducing RANKL expression. Interestingly, IL-17 is reported to upregulate MYC in some cancer cells [47, 48]. IL-1β, TNF-α, and LPS are known to have potent effects on the invasiveness of RA-FLS. In human neurons, GLS1 expression was upregulated by IL-1β or TNF-α treatment, and in a rat hepatoma model IL-6 or TNF-α treatment indirectly upregulated c-MYC expression [49, 50]. However, we did not observe upregulation of GLS1 by IL-1β, TNF-α, or LPS. Although the mechanism is unclear, IL-17-specific upregulation of GLS1 might be a cellular characteristic of RA-FLS, and suggests a novel role for IL-17 in RA-FLS and the pathogenesis of RA.

Since both glycolysis and glutaminolysis are associated with T-cell activation [51], GLS1 inhibition may also affect the immune system. Th17 cells and Treg cells show contrasting characteristics in terms of cellular metabolism; Th17 cells rely mainly on glycolysis [52], whereas Treg cells depend on glycolysis and fatty acid oxidation (FAO), and oxidative phosphorylation [53]. As Th17 cells and Treg cells are known to be two key players in the onset and maintenance of autoimmune arthritis in SKG mice [54], we studied the effect of GLS1 inhibition on Th17 and Treg cells. However, we found no difference in the number of Th17 cells and a decrease in the number of Treg cells in the spleens from compound 968- versus DMSO-treated SKG mice, suggesting that GLS1 inhibition minimally affected immune cells in our experimental system.

Several limitations of this study should be acknowledged. First, although we have revealed the critical role of GLS1 on cell proliferation of RA-FLS, we did not demonstrate the role of GLS1 on the invasive phenotype of RA-FLS. Second, we found that GLS1 mRNA was upregulated after cytokine stimulation, but we could not confirm the upregulation of GLS1 at the protein level. Third, we did not illustrate a precise molecular mechanism that is regulated by glutaminolysis in RA-FLS. To address this point, we examined the expression of c-MYC by immunoblotting (Additional file 9: Figure S8). Although there was no significant difference in c-MYC expression between RA-FLS and OA-FLS, we found that c-MYC expression tended to be higher in RA-FLS than in OA-FLS. Taken together, our in vitro and in vivo experiments suggest that glutaminolysis is activated in RA-FLS and that blocking glutaminolysis may be a novel therapeutic strategy for RA.

## Conclusions

In this study, we found that GLS1 expression was upregulated in RA-FLS, and that RA-FLS cell growth was decreased under glutamine-deprived, but not glucose-deprived, conditions. We also found that GLS1 inhibition suppressed RA-FLS proliferation and ameliorated inflammatory arthritis in SKG mice. These results suggest that glutamine metabolism is involved in the pathogenesis of RA and that GLS1 plays an important role in regulating RA-FLS proliferation, and may be a novel therapeutic target for RA.

## Additional files

Additional file 1: Table S1. Primer sequences used in this study. All primer sets were used for measuring gene expression by real-time PCR using SYBR® green chemistry. (DOCX 29 kb)

Additional file 2: Figure S1. GLS1 plays a major role in glutamine metabolism in FLS. GLS1 and GLS2 mRNAs were examined in 12 OA-FLS and 19 RA-FLS by real-time PCR, and the levels were normalized to that of GAPDH mRNA. Each experiment was performed in triplicate. Bars indicate mean ± SEM. (TIF 2028 kb)

Additional file 3: Figure S2. Glutamine/glutamate ratio was not significantly different between OA-FLS and RA-FLS. Intracellular glutamine/glutamate ratio in 7 OA-FLS and 11 RA-FLS were analyzed by GC/MS, and in 3 OA-FLS and 3 RA-FLS were analyzed by CE-MS. Bars indicate mean ± SEM. (TIF 2028 kb)

Additional file 4: Figure S3. siRNA efficiency of HK2, MCT4, GLS1, and PDK1 in RA-FLS. After transfection with HK2, MCT4, PDK1, GLS1, or control siRNA, mRNA levels were examined by real-time PCR in RA-FLS (*n* = 3 for HK2, MCT4, and GLS1, *n* = 4 for PDK1). Each experiment was performed in triplicate. Bars indicate mean ± SEM. *P < 0.05, **P < 0.01. (TIF 2028 kb)

Additional file 5: Figure S4. Silencing of MCT4, PDK1, or GLS1 did not significantly affect IL-6 or MMP-3 production in supernatants. After silencing of MCT4, PDK1, or GLS1, IL-6 and MMP-3 levels in culture supernatants of RA-FLS were examined by ELISA (*n* = 4). Each experiment was performed in duplicated. Bars indicate mean ± SEM. *P < 0.05. (TIF 2028 kb)

Additional file 6: Figure S5. PDGF stimulation did not enhance the proliferation of RA-FLS under the glutamine-deprived medium condition. RA-FLS, culturing in medium with or without glutamine (Gln), were stimulated with or without PDGF (10 ng/ml). RA-FLS proliferation was determined using BrdU assay 48 h after stimulation (*n* = 4). Each experiment was performed in quintuplicate. Bars indicate mean ± SEM. **P < 0.01. (TIF 2028 kb)

Additional file 7: Figure S6. IL-17 or PDGF stimulation did not significantly change the intracellular glutamine and glutamate levels in RA-FLS. Relative levels of intracellular glutamine and glutamate in 3 RA-FLS after IL-17 or PDGF stimulation were analyzed by GC/MS. Bars indicate mean ± SEM. (TIF 677 kb)

Additional file 8: Figure S7. Influence of GLS1 inhibition on immune cells was minimal in arthritic SKG mice. The number of RORγt-expressing CD4^+^ Th17 cells and FoxP3-expressing CD4^+^ regulatory T (Treg) cells in the spleens from compound 968-treated and DMSO-treated SKG mice at day 42 after ZyA injection were analyzed by flow cytometry (*n* = 5 for Th17 cells in DMSO group, *n* = 4 for Treg cells in DMSO group, and *n* = 4 for Th17 cells and Treg cells in compound 968 group). (TIF 2028 kb)

Additional file 9: Figure S8. c-MYC expression in RA-FLS. c-MYC protein levels in 5 OA-FLS and 5 RA-FLS were determined by immunoblotting. Protein levels were calculated by phosphorimager analysis. Bars indicate mean ± SEM. (TIF 677 kb)

## Abbreviations

APC/C: Anaphase promoting complex/cyclosome
CE-MS: Capillary electrophoresis-mass spectrometry
DMEM: Dulbecco’s modified Eagle’s medium
DMSO: Dimethyl sulfoxide
ELISA: Enzyme-linked immunosorbent assay
FAO: Fatty acid oxidation
FBS: Fetal bovine serum
FLS: Fibroblast-like synoviocytes
GAPDH: Glyceraldehyde-3-phosphate dehydrogenase
GC/MS: Gas chromatography-mass spectrometry
GLS: Glutaminase
HK: Hexokinase
IL: Interleukin
LPS: Lipopolysaccharide
MCT4: Monocarboxylate transporter
MMP: Matrix metalloproteinase
OA: Osteoarthritis
PCR: Polymerase chain reaction
PDGF: Platelet-derived growth factor
PDK: Pyruvate dehydrogenase kinase
RA: Rheumatoid arthritis
sIL-6R: Soluble interleukin-6 receptor
siRNA: Small interfering RNA
TCA: Tricarboxylic acid
TNF: Tumor necrosis factor
Treg: Regulatory T
ZyA: Zymosan A
