# Supplementary figures and images for: Glutaminase 1 plays a key role in the cell growth of fibroblast-like synoviocytes in rheumatoid arthritis
Source: Arthritis Res Ther. 2017 Apr 11;19:76. doi: 10.1186/s13075-017-1283-3 (PMC5387190; doi:10.1186/s13075-017-1283-3)

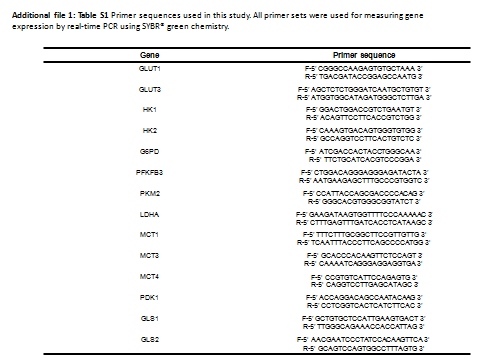

Supplement: Supplementary file 1 — Primer sequences used in this study. All primer sets were used for measuring gene expression by real-time PCR using SYBR® green chemistry. (DOCX 29 kb) [file 13075_2017_1283_MOESM1_ESM.docx]

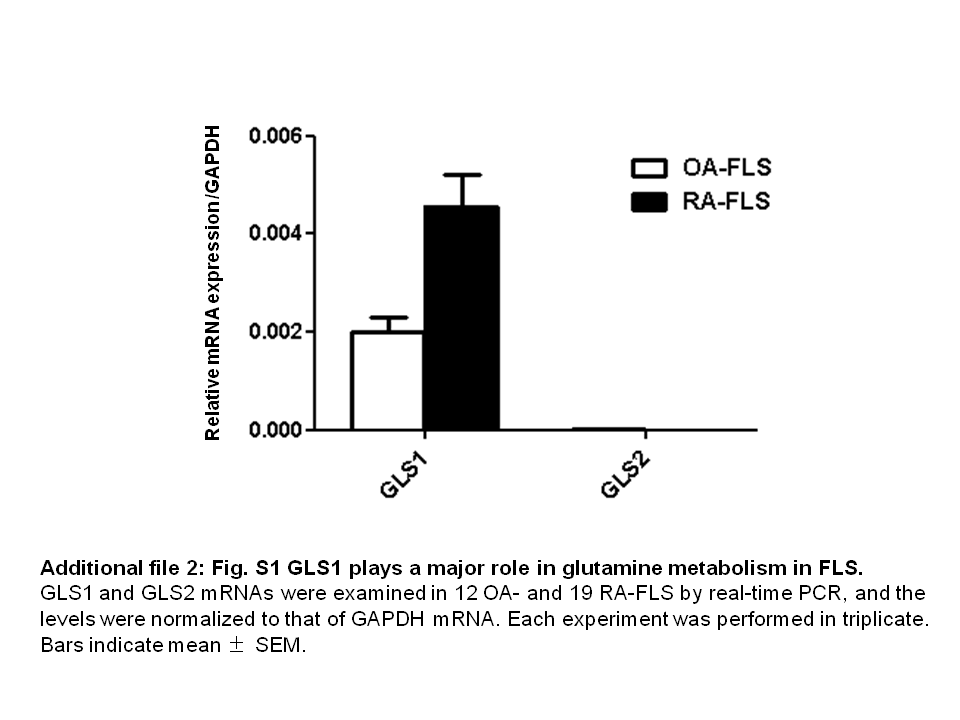

Supplement: Supplementary file 2 — GLS1 plays a major role in glutamine metabolism in FLS. GLS1 and GLS2 mRNAs were examined in 12 OA-FLS and 19 RA-FLS by real-time PCR, and the levels were normalized to that of GAPDH mRNA. Each experiment was performed in triplicate. Bars indicate mean ± SEM. (TIF 2028 kb) [file 13075_2017_1283_MOESM2_ESM.tif]

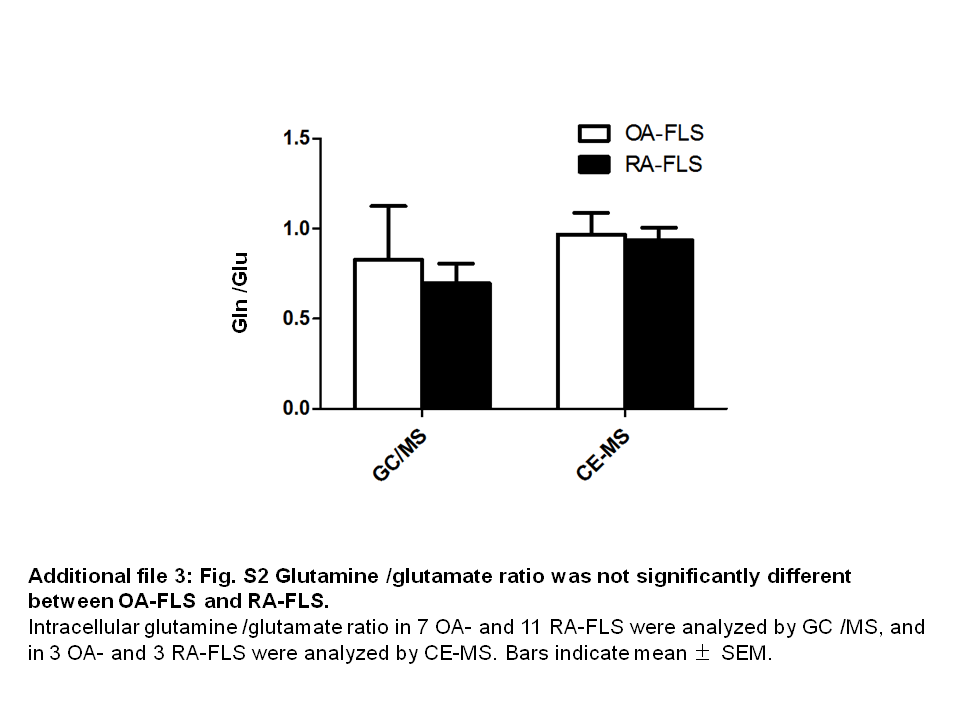

Supplement: Supplementary file 3 — Glutamine/glutamate ratio was not significantly different between OA-FLS and RA-FLS. Intracellular glutamine/glutamate ratio in 7 OA-FLS and 11 RA-FLS were analyzed by GC/MS, and in 3 OA-FLS and 3 RA-FLS were analyzed by CE-MS. Bars indicate mean ± SEM. (TIF 2028 kb) [file 13075_2017_1283_MOESM3_ESM.tif]

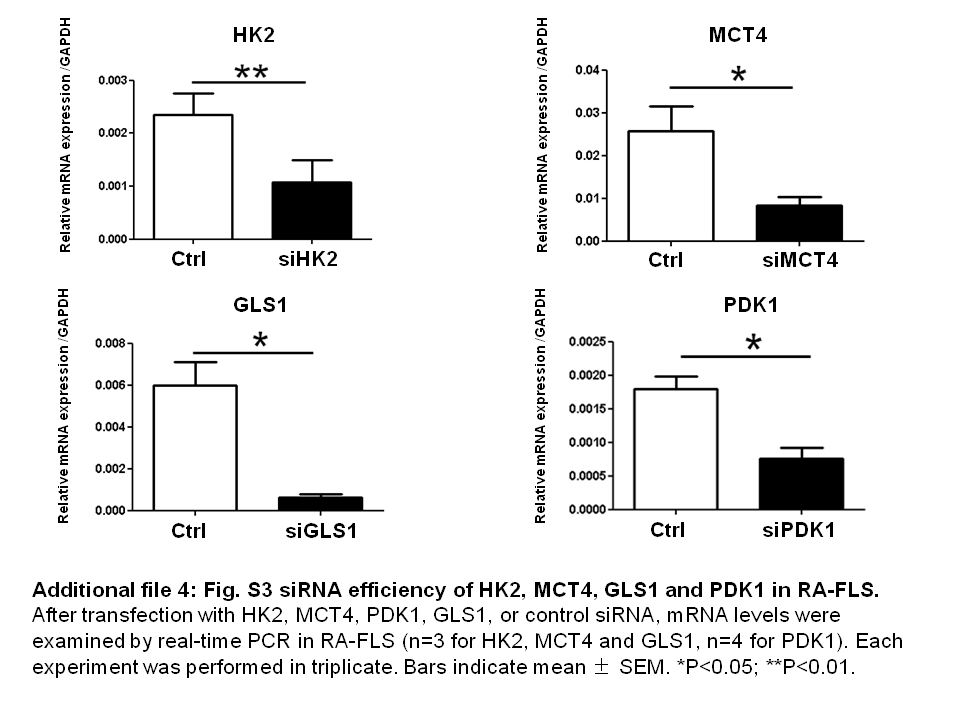

Supplement: Supplementary file 4 — siRNA efficiency of HK2, MCT4, GLS1, and PDK1 in RA-FLS. After transfection with HK2, MCT4, PDK1, GLS1, or control siRNA, mRNA levels were examined by real-time PCR in RA-FLS (n = 3 for HK2, MCT4, and GLS1, n = 4 for PDK1). Each experiment was performed in triplicate. Bars indicate mean ± SEM. *P < 0.05, **P < 0.01. (TIF 2028 kb) [file 13075_2017_1283_MOESM4_ESM.tif]

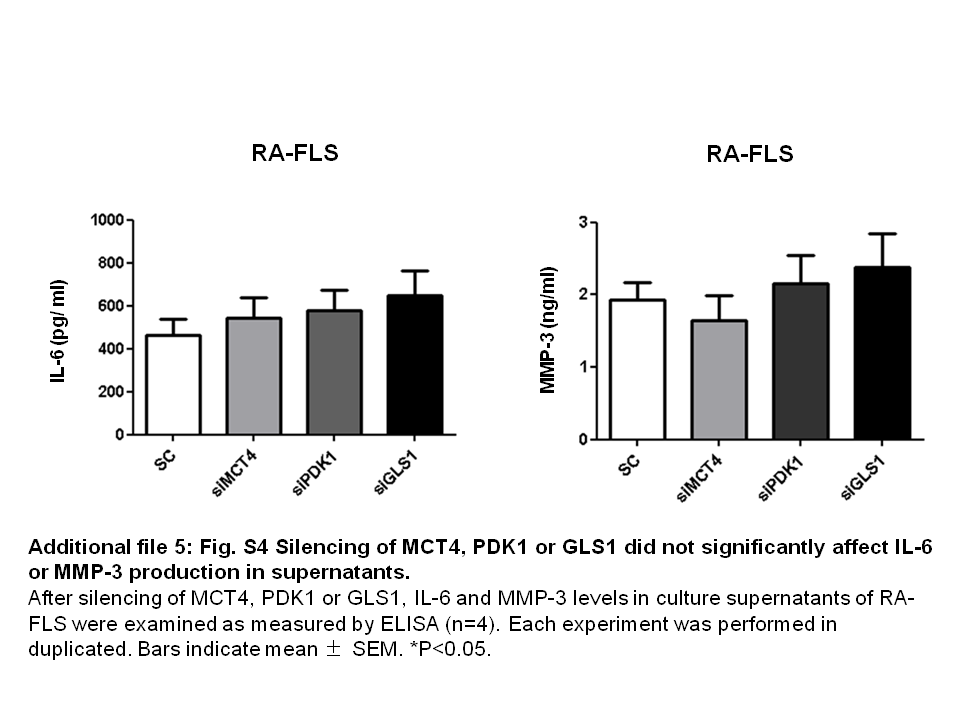

Supplement: Supplementary file 5 — Silencing of MCT4, PDK1, or GLS1 did not significantly affect IL-6 or MMP-3 production in supernatants. After silencing of MCT4, PDK1, or GLS1, IL-6 and MMP-3 levels in culture supernatants of RA-FLS were examined by ELISA (n = 4). Each experiment was performed in duplicated. Bars indicate mean ± SEM. *P < 0.05. (TIF 2028 kb) [file 13075_2017_1283_MOESM5_ESM.tif]

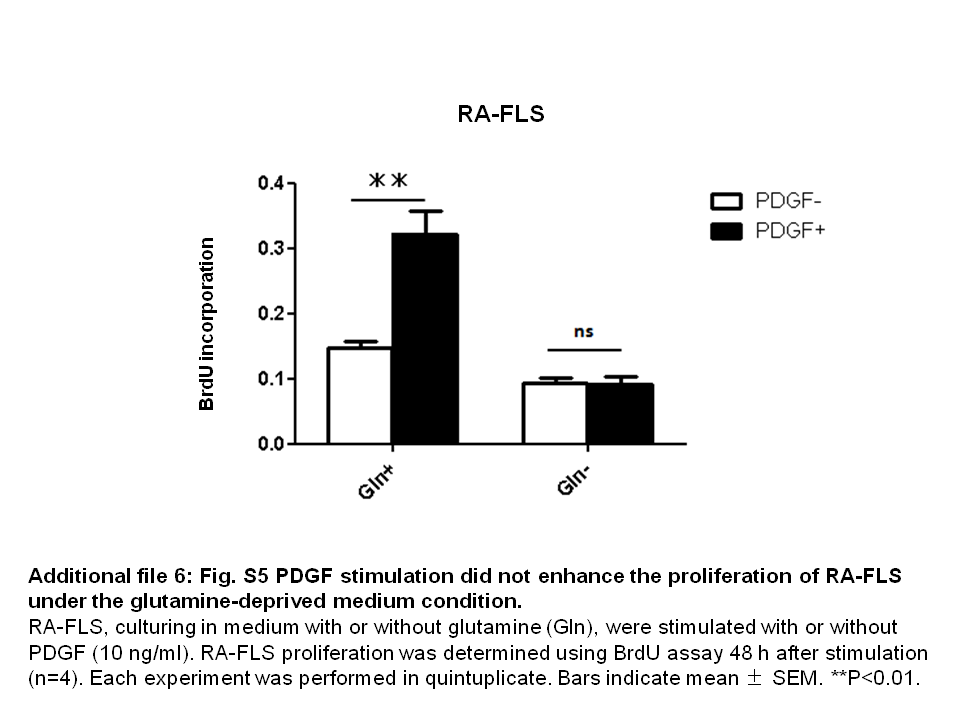

Supplement: Supplementary file 6 — PDGF stimulation did not enhance the proliferation of RA-FLS under the glutamine-deprived medium condition. RA-FLS, culturing in medium with or without glutamine (Gln), were stimulated with or without PDGF (10 ng/ml). RA-FLS proliferation was determined using BrdU assay 48 h after stimulation (n = 4). Each experiment was performed in quintuplicate. Bars indicate mean ± SEM. **P < 0.01. (TIF 2028 kb) [file 13075_2017_1283_MOESM6_ESM.tif]

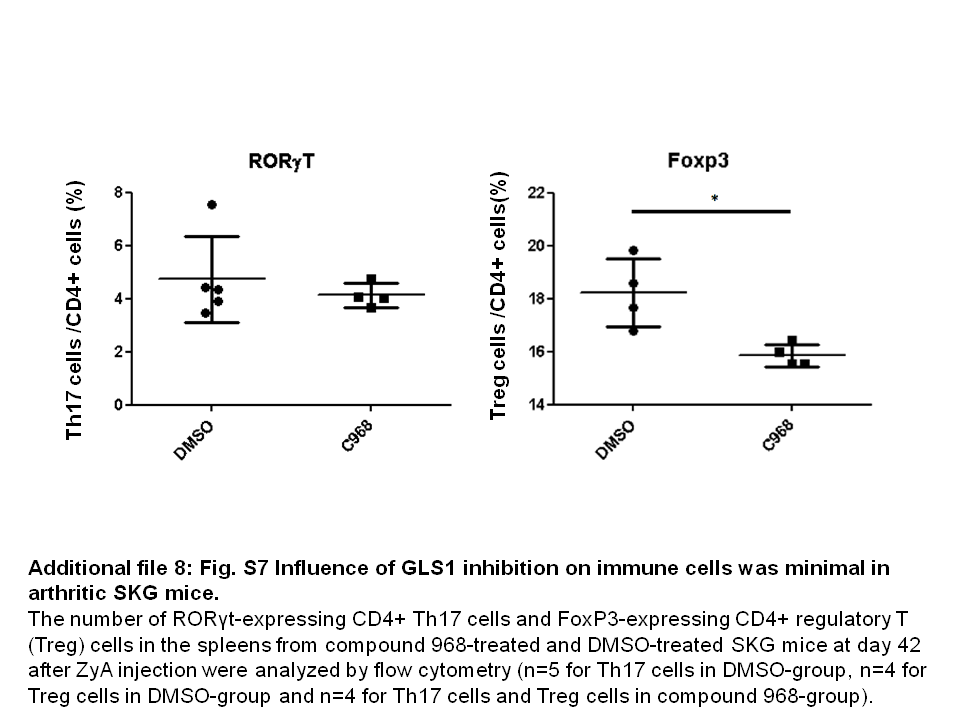

Supplement: Supplementary file 8 — Influence of GLS1 inhibition on immune cells was minimal in arthritic SKG mice. The number of RORγt-expressing CD4+ Th17 cells and FoxP3-expressing CD4+ regulatory T (Treg) cells in the spleens from compound 968-treated and DMSO-treated SKG mice at day 42 after ZyA injection were analyzed by flow cytometry (n = 5 for Th17 cells in DMSO group, n = 4 for Treg cells in DMSO group, and n = 4 for Th17 cells and Treg cells in compound 968 group). (TIF 2028 kb) [file 13075_2017_1283_MOESM8_ESM.tif]
